# Safety and Practicalities of Bispecific T‐Cell Engager Administration in a District General Hospital Setting

**DOI:** 10.1002/jha2.70074

**Published:** 2025-06-06

**Authors:** Benjamin Lau, Simran Gabrie, Prudence Hobbs, Joel Newman, Abier Elzein, Nigel Sargant, Anna Cowley, Albena Nikolova, Cliona Flanagan, Lorraine Burt, Maggie Saddleton, Clare Evans, Theresa Street, Hiba Mahbak, Arturo Lazaro, Richard Grace, John R. Jones

**Affiliations:** ^1^ Department of Haematology Eastbourne District General Hospital East Sussex Healthcare NHS Trust Eastbourne UK; ^2^ Brighton and Sussex Medical School Brighton UK; ^3^ Department of Haematological Medicine King's College Hospital King's College Hospital NHS Foundation Trust London UK

1

The therapeutic landscape for multiple myeloma has changed drastically over recent years, leading to improvements in outcomes [1]. Bispecific T‐cell engager (BiTE) therapy is one such advancement that promotes T‐cell mediated cytotoxicity towards plasma cells by binding to both CD3 and plasma cell‐specific antigens [2]. Although these have shown promising results in the relapsed/refractory setting, adverse effects are well described and can be similar to those of CAR‐T therapy, including cytokine release syndrome (CRS) and immune effector cell‐associated neurotoxicity syndrome (ICANS) [3]. Consequently, the initiation of BiTEs in centres without experience of managing these complications in the context of CAR‐T has been limited. In early‐phase BiTE studies however, Grade 3 or greater CRS or ICANS were rare, noted in < 1% of patients [4, 5, 6], much lower than the incidence with CAR‐T [7]. Therefore, with comprehensive management algorithms, the treatment could be considered deliverable in non‐tertiary centres. We hence describe our experience of delivering BiTE therapies to a cohort of 11 patients in a District General Hospital setting, with a focus on safety and feasibility.

Between January 2023 and September 2024, 11 patients received treatment with BiTEs including teclistamab (*n* = 3), talquetamab (*n* = 1) and elranatamab (*n* = 7) for triple‐class exposed relapsed/refractory multiple myeloma at Eastbourne District General Hospital. The cohort also included a heavily pre‐treated population, with a median of 5 prior lines of therapy, which were 100% triple‐class refractory and 64% penta‐refractory. 9 of the 11 patients (82%) had been treated with pomalidomide (Table 1). Individual patient characteristics are detailed in Table S1. All patients received dose escalations in line with the trial protocols of the Majes‐TEC1, MonumenTAL‐1 and MagnetisMM‐3 early‐phase trials for teclistamab, talquetamab, and elranatamab, respectively [4, 5, 6].

**TABLE 1:** Baseline characteristics of patients receiving BiTE therapy at Eastbourne District General Hospital, as well as CRS rate and overall response.

Patient characteristics	
Patient number	11
BCMA/GPRC5D target, *n*	10/1
Prior BCMA targeted therapy, *n*	2
Median prior lines of therapy (range)	5 (3–11)
Median age	76 (range 56–78)
Male, *n* (%)	7 (64)
Race, *n* (%)	
White	10 (91)
Black	1 (9)
ECOG performance status *n* (%)	
0	1 (9)
1	8 (73)
2	2 (18)
Type of myeloma *n* (%)	
IgG	4 (36)
IgA	4 (36)
Light chain	2 (18)
Oligosecretory	1 (9)
ISS (at diagnosis) *n* (%)	
I	6 (55)
II	1 (9)
III	1 (9)
Unknown	3 (27)
Cytogenetic risk (at diagnosis) *n* (%)	
Standard	2 (18)
High*	4 (36)
Not known/failed	5 (45)
* t(4;14), t(14;16), del(17p), gain/amp(1q)	
Extramedullary disease *n* (%)	5 (45)
Prior stem cell transplant *n* (%)	5 (45)
Treatment refractory status *n* (%)	
Triple	11 (100)
Penta	7 (64)
Pomalidomide treated	9 (82)

**Response and CRS rate**	
Best response *n* (%)	
CR	1 (9)
VGPR	3 (27)
PR	1 (9)
SD	0
Refractory	6 (55)
CRS	
No CRS	7 (64)
Grade 1	4 (36)
Grade ≥ 2	0

*Note*: ISS is determined by International Myeloma Working Group Criteria [8]. Triple class refractory is defined as refractoriness to proteasome inhibitor, immunomodulatory drug (IMiD), and anti‐CD38 antibody. Penta‐refractory is defined as refractoriness to lenalidomide, pomalidomide, bortezomib, carfilzomib, and anti‐CD38 antibody.

Due to the risk of immune‐effector syndromes (IESs), a standard operating procedure (SOP) document was prepared prior to commencing therapy. The SOP included methods of administration, monitoring guidance, summary of the symptoms and grading of CRS and ICANS according to ASTCT consensus [9], treatment algorithms and clear indications for escalation to intensive care. Tocilizumab was also made available on the ward prior to administration and additional supply could be dispensed at short notice from pharmacy. The attending consultant was informed of treatment initiation and reviewed patients prior to the first dose. They were also informed if CRS or ICANS of any grade developed, including out of hours. At our centre, patients were monitored as inpatients for a minimum of 48 h following each dose escalation. Due to the dose escalations on days 1, 4, and 7, patients remained inpatients for an average of 9 days. At discharge, patients were counselled on symptoms to be aware of, as well as given a thermometer, emergency contacts, alert card, and educational materials.

All 11 patients completed the dose‐escalation phase and at least one full cycle of therapy. Grade 1 CRS with a temperature of ≥ 38°C was seen in four (35%) patients and no patients developed CRS of Grade 2 or above (Figure 1A). These patients were also managed according to local guidelines for fever in immunocompromised patients, including the use of antibiotics, and all patients were escalated to the attending Consultant in line with our SOP. No patients required tocilizumab but it was available if fever persisted or progression to CRS Grade 2 or above was noted. All patients were managed on the ward, with no input by the intensive care team required, and we did not see evidence of ICANS in any patient.

**FIGURE 1:**
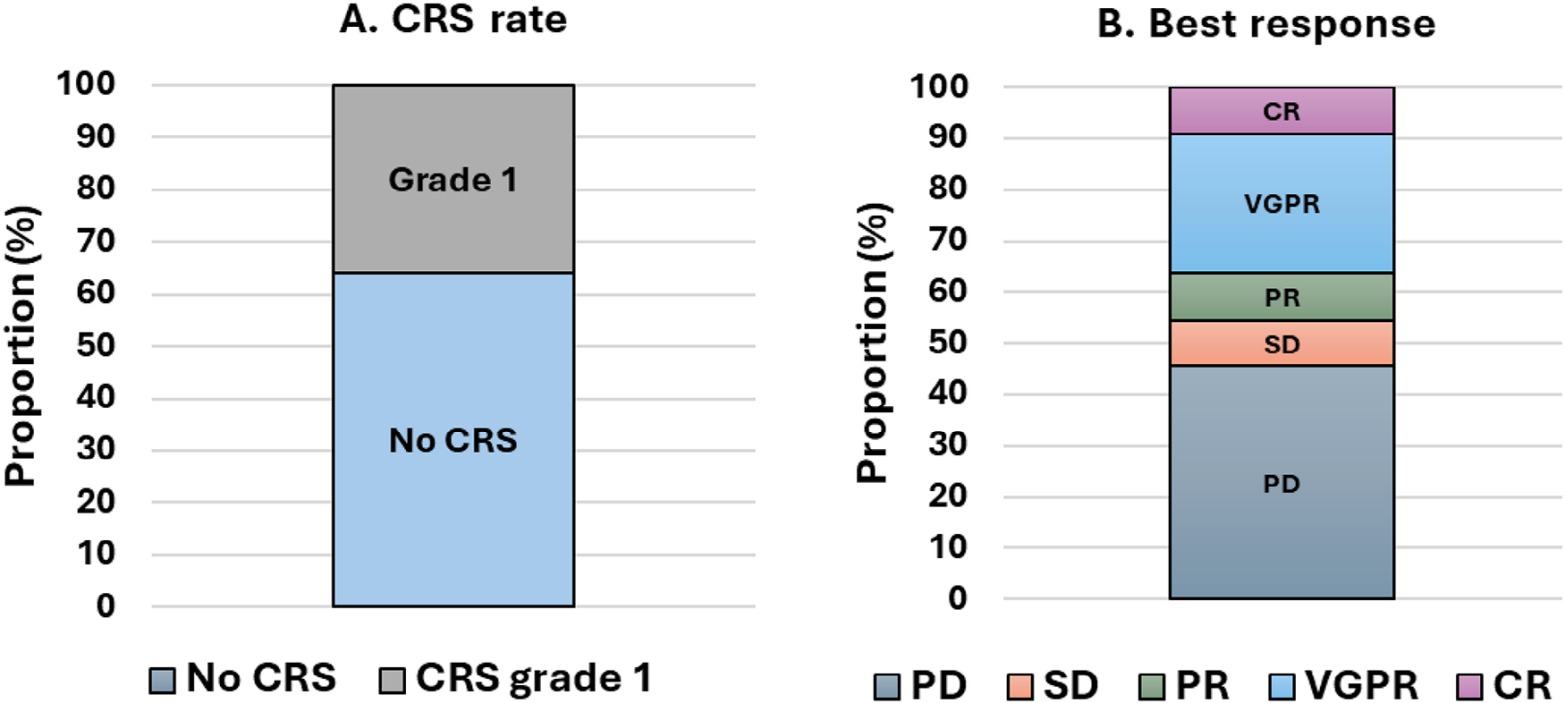
Outcomes of BiTE therapy in patients receiving either teclistamab, talquetamab or elranatamab at Eastbourne District General Hospital. (A) Incidence and grade of CRS whilst receiving BiTE therapy. (B) Best response rate according to the International Uniform Response Criteria [10]. Three patients are receiving ongoing therapy. CR; complete response, CRS; cytokine release syndrome, PD; progressive disease, PR; partial response, SD; stable disease, VGPR; very good partial response.

Although incidence of IESs of Grade 3 and above was rare in early‐phase BiTE trials, CRS overall was common, seen in up to 80% of patients [4, 5, 6]. A total of 22.7% of patients also received tocilizumab for CRS or ICANS whilst receiving elranatamab [6]. The findings within our cohort therefore contrast with the trial findings, highlighting the importance of implementing comprehensive SOPs prior to administering these therapies, even if the complications are not seen. Step‐up dosing was also used, which has been shown to reduce the duration and intensity of CRS if it occurs [11] and may account for some of the differences.

Aside from IESs, infections were also common, including one Grade 5 infection, illustrating the potential immune compromise that can occur in patients receiving BiTE therapies, particularly in heavily pre‐treated individuals. The effect of long‐term administration on immune function should therefore also be considered, a phenomenon which is well‐described [12] and more long‐term data is required. We did note that the non‐involved light chain level was below the limit of detection in 10 (91%) of the 11 patients, suggesting marked compromise of the non‐malignant plasma cell reservoir. In our experience intravenous immunoglobulin has also been used in this context, although it was not required in our patient cohort. Otherwise, six other patients died during the period reported due to progressive/refractory disease, and no patient died as a direct result of the treatment. Other complications are outlined in Table S2.

Our objective response rate (ORR), as determined by achieving a partial response (PR) or better, was noted in five patients (45%) with six patients being refractory to BiTE therapy (Figure 1B). This is less than that reported in the trials, but despite the responses being inferior the ORR is favourable when compared to other options used in the later stages of disease, including pomalidomide [13, 14, 15].

We therefore suggest that administration of BiTEs for relapsed/refractory myeloma may be undertaken in non‐tertiary centres as long as robust SOPs and management protocols are created and adhered to, and widespread education about recognition and management of complications is carried out. This is important as the use of BiTEs and other immune‐effector strategies is likely to become universal across multiple cancer types, meaning that this non‐tertiary experience will be required. In our cohort, patients received a median of five prior lines of therapy. We would emphasise that additional caution is required if BiTEs are used earlier in the treatment algorithm, when the T‐cell repertoire is likely to be fully functional and the disease burden high. In this population of patients, CRS and ICANS may theoretically be more marked and new data and experience must be collated in this regard.

## Author Contributions

B.L., J.J. and S.G. collected and analysed the data, generated the figures and tables, and co‐wrote the manuscript. P.H., R.G., A.L. and J.J. devised the treatment pathway and BiTE standard operating procedure. All authors were involved in patient care, discussed the results, and commented on the final manuscript.

## Conflicts of Interest

The authors declare no conflicts of interest.

## Supporting information




**Supplementary Table 1**. Characteristics of individual patients receiving BiTE therapy at Eastbourne District General Hospital, including demographics and prior lines of treatment. **Supplementary Table 2**. Adverse events noted in >50% of patients.

## Data Availability

Due to patient confidentiality, the datasets used in this report have not been made publicly available. However, upon reasonable request, certain subsets of anonymised data may be made accessible.
