# Supplementary material for: Safety and Practicalities of Bispecific T‐Cell Engager Administration in a District General Hospital Setting
Source: EJHaem. 2025 Jun 6;6(3):e70074. doi: 10.1002/jha2.70074 (PMC12141921; doi:10.1002/jha2.70074)
Supplement: Supplementary file 1 — Supplementary Table 1. Characteristics of individual patients receiving BiTE therapy at Eastbourne District General Hospital, including demographics and prior lines of treatment. Supplementary Table 2. Adverse events noted in >50% of patients. [file JHA2-6-e70074-s001.docx]

| Supplementary Table 1 | | | | | | | | | | | |
| --- | --- | --- | --- | --- | --- | --- | --- | --- | --- | --- | --- |
| Patient | **1** | **2** | **3** | **4** | **5** | **6** | **7** | **8** | **9** | **10** | **11** |
| Age | 78 | 74 | 74 | 56 | 77 | 68 | 77 | 78 | 72 | 75 | 61 |
| Sex | F | M | M | M | F | M | M | F | M | M | F |
| ECOG | 1 | 1 | 1 | 1 | 1 | 2 | 2 | 1 | 1 | 1 | 0 |
| Type | IgA K | IgA K | Kappa (IgG) | IgG L | IgG K | kappa (IgA) | IgA K | IgG L | Oligosecretory | IgG k | IgA L |
| R-ISS | Not known | 1 | 1 | Not known | 1 | 1 | Not known | 2 | 1 | 3 | 1 |
| Geographic region | Caucasian | Caucasian | Caucasian | Caucasian | Caucasian | Caucasian | Caucasian | Afro-Caribbean | Caucasian | Caucasian | Caucasian |
| Triple class refractory? | Y | Y | Y | Y | Y | Y | Y | Y | Y | Y | Y |
| Penta-drug exposed? | N | Y | Y | Y | N | Y | N | Y | Y | N | Y |
| Penta-drug refractory? | N | Y | Y | Y | N | Y | N | Y | Y | N | Y |
| Pomalidomide exposed | Y | Y | Y | Y | Y | Y | Y | Y | Y | N | N |
| Prior lines | 4 | 5 | 6 | 11 | 9 | 4 | 4 | 6 | 7 | 3 | 5 |

**Supplementary data**

**Supplementary Table 1.** Characteristics of individual patients receiving BiTE therapy at Eastbourne District General Hospital, including demographics and prior lines of treatment.

| **Supplementary Table 2** | | |
| --- | --- | --- |
| **Adverse Event** | **All grade n (%)** | **Grade 3 or above n (%)** |
| **Cytopenia**   - **Anaemia** - **Thrombocytopenia** - **Neutropenia** | **11 (100)**  **8 (73)**  **8 (73)** | **5 (45)**  **4 (36)**  **5 (45)** |
| **Infection** | **6 (55)** | **5 (45)** |
| **Alkaline phosphatase** | **7 (64)** | **1 (9)** |

**Supplementary Table 2.** Adverse events noted in >50% of patients
